# Child-Centrism as an Adaptive Parenting Style: A Prospective Study in Motherhood from Women’s Life Satisfaction Including Cognitive–Emotional Resources

**DOI:** 10.3390/children12050601

**Published:** 2025-05-05

**Authors:** Celia Arribas, Patricia Catalá, Cecilia Peñacoba, Maria Crespo, Miri Kestler-Peleg

**Affiliations:** 1 Department of Psychology, Rey Juan Carlos University, Avda. de Atenas s/n, 28922 Alcorcón, Madrid, Spain; c.arribasj.2020@alumnos.urjc.es (C.A.); cecilia.penacoba@urjc.es (C.P.); maria.crespo.diaz@urjc.es (M.C.); 2 School of Social Work, Ariel University, Emek Dotan 1, Kochav Yair 40700, Israel; mirikp@ariel.ac.il

**Keywords:** child-centrism, women’s life satisfaction, maternal self-efficacy, positive affect, parenting style, motherhood, prospective

## Abstract

Background: Child-centrism refers to a parenting style where parents prioritize children’s needs above their own. Early research suggested that it could harm parental well-being, yet recent findings indicate that mothers adopting this style may experience greater psychological well-being and meaning in child-rearing. This study examines child-centrism as a complex, context-dependent style and aims to clarify its adaptive or non-adaptive nature through a prospective, longitudinal approach. Methods: A four-wave design included 231 mothers assessed at (T1) third trimester of pregnancy, (T2) eight weeks postpartum, (T3) five months postpartum, and (T4) five years postpartum. Self-reported measures included life satisfaction (T1), maternal self-efficacy (T2), positive affect (T3), and child-centrism (T4). Sociodemographic data (age, family status, education, income, working) were also collected. Statistical analyses tested a serial mediation model. Results: Life satisfaction at T1 significantly predicted maternal self-efficacy at T2 (*p* = 0.002), which in turn enhanced positive affect at T3 (*p* < 0.001). Positive affect at T3 then positively influenced child-centrism at T4 (*p* = 0.023). However, life satisfaction did not directly affect child-centrism (*p* = 0.970), indicating a sequential mediation pathway rather than a direct link. Conclusions: These findings highlight that higher prenatal life satisfaction indirectly fosters an adaptive form of child-centrism through maternal self-efficacy and positive affect. Integrating motherhood into a woman’s sense of identity appears to be a protective factor, promoting healthier cognitive and affective parenting resources. Preventive interventions could focus on strengthening women’s life satisfaction and self-efficacy early in pregnancy, thereby enhancing affective well-being and potentially leading to a more meaningful, child-centric parenting style. Further research should include broader maternal and child well-being indicators.

## 1. Introduction

In the field of parenting, the concept of child-centrism refers to a psychological mindset in which parents place their child at the center of their lives, prioritizing the child’s needs over their own and dedicating significant emotional, attentional, temporal, and economic resources [1]. Unlike helicopter parenting or maternal overinvolvement, which are characterized by intrusive and controlling behaviors that limit children’s autonomy [2,3,4], child-centrism reflects an emotional and cognitive orientation toward the child’s well-being without necessarily restricting independence. Historically, following World War II, the rise of developmental psychology heightened the emphasis on maternal responsibility for child well-being. This shift led to the proliferation of child-centered parenting recommendations [5,6] that persisted through the 1990s, as social institutions increasingly educated parents about the importance of spending quality time with their children [5,7].

Pregnancy constitutes a pivotal developmental period for maternal identity formation and subsequent child-centrism [8], conceptualized “maternal preoccupation” as a distinct psychological state emerging during pregnancy, which redirects women’s focus toward the developing infant and their future maternal role [9]. This psychological reorientation establishes foundations for later child-centered parenting. Research demonstrates that prenatal maternal–fetal attachment correlates with enhanced postnatal parental sensitivity and child-prioritization behaviors [10,11].

While child-centrism may foster positive outcomes, such as increased involvement and responsiveness, it has also been linked to maladaptive parenting styles—such as the “tiger mother” phenomenon or helicopter parenting—which can contribute to elevated stress, fatigue, anxiety, and guilt [1,2,3,4,5]. Recent studies, however, have begun to challenge this negative view, suggesting that a child-centric approach may actually be associated with greater happiness and life meaning among parents [1]. Indeed, maternal life satisfaction appears to be strongly linked to child development outcomes [12], with life satisfaction defined as the degree to which an individual evaluates their life against their own standards [13].

Various factors influence life satisfaction during pregnancy, including maternal age, socioeconomic and educational levels, and the number of children [14]. Social support during pregnancy has emerged as a critical determinant of well-being [15], whereas adverse experiences such as abortion can temporarily diminish life satisfaction [16]. Moreover, higher life satisfaction has been linked to greater maturity in future parenting and to stronger prenatal attachment [17,18,19].

In the postpartum period, maternal well-being is further influenced by factors such as maternal self-efficacy and positive affect. Maternal self-efficacy—defined as a mother’s perception of her competence in fulfilling her maternal role [20]—is crucial for creating a nurturing home environment, strengthening parent–child relationships, and fostering adaptive behaviors in children [21]. Research has shown that self-efficacy tends to increase after childbirth [22,23], marking this period as critical for its development [24]. Additionally, positive affect—defined as the degree to which an individual feels enthusiastic, active, and alert [25]—has been associated with secure attachment patterns and greater responsiveness to a child’s needs [26,27,28,29,30,31].

Although previous studies [20,25,26,27,28,29,30] have examined these variables independently, there remains a significant gap in research exploring the longitudinal relationships among prenatal life satisfaction, maternal self-efficacy, positive affect, and the adoption of a child-centric parenting style during the preschool years. The present study aims to fill this gap by investigating these interrelated constructs over time.

This particular research is carried out in Israel. Motherhood in this country is conceived as a central component of female identity and a distinct social and national value [32]. Although empirical research has documented how Israel’s pronatalist policies have established motherhood as a central life goal [33,34], the ways in which these policies shape maternal practices after childbirth have not been sufficiently explored. Nevertheless, it can be reasonably assumed that the elevation of motherhood as a national and social ideal, combined with strong cultural expectations for intensive investment in childrearing, contributes to the development of child-centered parenting norms among Jewish Israeli mothers. Israeli culture promotes an ideology of “intensive motherhood”, wherein women are expected to invest the majority of their emotional, cognitive, and social resources in raising their children [35]. Within a social context characterized by familism and pronatalist policies, motherhood is also imbued with symbolic and national significance [36]. This emphasis on natality is heightened by Israel’s geopolitical situation, surrounded by several hostile states, which has historically encouraged population growth as a means of ensuring demographic strength against neighboring threats. The social pressure to achieve parental success in Israel, built upon values of both individual and collective excellence, further intensifies the tendency toward child-centrism [37]. Within this reality, motherhood is not merely a personal role but is perceived as a collective mission, where the child’s needs are positioned at the center of parental practice and identity.

Specifically, taking into account the above contextual variables, the present research hypothesizes the following: (1) High life satisfaction in women during pregnancy is essential for child-centrism as an adaptive parenting style; (2) A sequence of mediating processes between life satisfaction in pregnancy and child-centrism as a parenting style are proposed: maternal role self-efficacy and positive affect. Specifically, life satisfaction generates higher maternal self-efficacy, which in turn contributes to positive affect, ultimately affecting child-centrism. This comprehensive approach will deepen our understanding of the mechanisms underlying child-centrism and inform the development of targeted interventions to support a healthy transition into motherhood.

## 2. Materials and Methods

### 2.1. Participant and Procedure

The present study is a prospective longitudinal study with four measurement points (at the third trimester of pregnancy, eight weeks after birth, five months after birth, and five years after birth). A total of 231 Jewish Israeli mothers were recruited from eight cities in central Israel. This recruitment was conducted through the Israeli public health funds. Members of the research team identified and approached suitable candidates, briefed them on the study parameters, and solicited their voluntary participation. The administration of questionnaires occurred in participants’ residences, with completed forms being returned to the investigators through either conventional mail or electronic submission. We rigorously maintained participant anonymity by separating respondents’ personal identifiers from the database and assigning numerical codes for identification purposes.

A total of 549 pregnant women agreed to participate in the study. In the first phase (T1), during the third trimester of pregnancy, all participants completed questionnaires assessing life satisfaction and sociodemographic variables: age, education, income, planned pregnancy, and employment activity. In the second phase (T2), conducted eight weeks after delivery, 471 mothers completed maternal self-efficacy measures. Five months after delivery, 352 mothers participated in the third phase (T3), where positive affect was assessed. Finally, in the fourth phase (T4), conducted in 2014, 231 mothers completed child-centered approach measures. Given that recruitment and retention of this population are difficult [38,39], the study finally recorded a response rate of 42.07%, comparable to that obtained in other longitudinal studies [40,41,42]. Participant dropout was partly attributed to difficulties in following up with individuals, reluctance to share personal information, time constraints, and limited availability to participate. No statistically significant differences were observed in sociodemographic and outcome variables (i.e., life satisfaction, maternal self-efficacy, and positive affect) between the sample who did not participate in all the temporal moments (n = 549) and the one that finally concluded the study (n = 231).

The ethics committee of the four Israeli Health Funds and the ethics committee of Tel Aviv University approved this study.

### 2.2. Measures

#### 2.2.1. Life Satisfaction (Time 1)

Life satisfaction was measured using the Satisfaction with Life Scale (SWLS) [13], which consists of five items designed to assess a person’s overall evaluation of life (e.g., “In most respects, my life is close to my ideal”). Participants responded on a 7-point scale ranging from 1 (strongly disagree) to 7 (strongly agree), and the overall score was obtained by averaging the responses to each item (theoretical range 1–7). Higher scores indicate greater life satisfaction. In this study, the SWLS demonstrated good internal consistency (α = 0.858).

#### 2.2.2. Maternal Self-Efficacy (Time 2)

Maternal self-efficacy was assessed using the Sense of Competence in Parenting Questionnaire (PSOC) [43]. This 18-item measure assesses mothers’ perceptions of their competence in their parenting role. Responses were recorded on a 4-point Likert scale (1 = not at all, 4 = very much). The overall score is calculated as the average of all items (range 1–4), with higher scores reflecting greater self-efficacy. The scale showed acceptable reliability in this sample (α = 0.85).

#### 2.2.3. Positive Affect (Time 3)

The Positive and Negative Affect Scale (PANAS) [25] was used to assess positive affect. This instrument includes 10 items with responses on a 5-point Likert scale (1 = very little or not at all, 5 = extremely), reflecting the intensity of affect experienced over the past two weeks. Mean scores for positive affect (e.g., “happy”) were calculated (theoretical range 1–5), and the subscale showed excellent internal consistency (α = 0.86).

#### 2.2.4. Child-Centrism (Time 4)

Child-centrism was measured using the Child-Centrism Scale [1], which consists of 7 items rated on a 7-point Likert scale (0 = none to 6 = a lot) with the overall score calculated as the average of all items (range 1–7). This scale assesses the degree to which parents invest personal resources to the detriment of their own needs. The internal consistency of this measure was acceptable (α = 0.75). This scale was selected for its conceptual alignment with the study’s focus on parents’ emotional and cognitive orientation toward their child’s well-being, and for its proven suitability for longitudinal designs requiring concise yet reliable measures.

#### 2.2.5. Sociodemographic Data

This study also collected key covariates, including age (in years), education (total years of formal schooling), income (categorized relative to the average income in Israel as below average, approximately average, or above average), planned pregnancy (yes/no), and employment status (categorized as full-time employment, part-time employment, or not working).

The Hebrew versions of the SWLS [44], PSOC [45], and PANAS [46,47] have been widely used and validated in Israeli samples. Regarding the Child-Centrism Scale, the translation process followed standard guidelines, including independent translations by two parenting researchers, a back-translation by a bilingual expert, and a review of discrepancies. This Hebrew version has recently been employed with excellent reliability (α = 0.95) in an independent Israeli study [48]. In the present study, the scale showed acceptable internal consistency (α = 0.75).

### 2.3. Statistical Analysis

Analyses were performed using SPSS version 22 [49]. Initially, descriptive analyses were performed, along with internal consistency assessments using Cronbach’s alpha and Pearson’s correlation analysis. For continuous variables, means, standard deviations, and ranges (medians) were reported, while categorical data were expressed as frequencies and percentages. A significance level of *p* < 0.05 was set for all tests.

For serial multiple mediation analysis, the PROCESS macro for SPSS was used (model 6), considering two significant mediators. Following the recommendations of Hayes [50], regression coefficients are presented in an unstandardized format, as this allows for a more direct and substantive interpretation. A model was evaluated in which the predictor variable was life satisfaction in the third trimester of pregnancy, the first mediator was self-efficacy, the second mediator was positive affect, and the outcome variable was child-centrism. Statistical significance was defined as a two-sided *p* value of <0.01. To test statistical significance, the bootstrap method was used with 5000 bootstrap samples to construct 95% confidence intervals.

## 3. Results

### 3.1. Sample Characteristics

The participants’ ages ranged between 27 and 51, with the mean age of 37.10 years (*SD* = 4.34). Their educational background varied from 12 to 27 years of formal education, with an average of 16.46 (*SD* = 1.97). Most participants were married or living with a partner (96.1%), while the remainder were single (0.9%) and divorced or separated (3%). Regarding employment status, 60.2% of participants worked full-time, 21.7% worked part-time, and 18.1% were unemployed. In terms of economic status, 67.3% reported having above-average income in Israel, 23.6% reported an average income, and 9.1% reported a lower income compared to the national average. Lastly, in relation to family, 44.6% of participants had no children at the time of data collection, 27.7% had one child, 20.3% had two children, 5.2% had three children, and 2.2% had four or more children. Regarding pregnancy planning, 93% of participants reported that their pregnancy was planned, whereas 7% stated it was unplanned. Additionally, 89.6% of participants conceived naturally, while 10.4% conceived through assisted reproductive methods.

### 3.2. Descriptive Statistics and Pearson Correlation Analysis Among the Variables

Table 1 presents the descriptive statistics for four key variables measured at different time points and their intercorrelations. Life satisfaction had a mean of 5.43 (*SD* = 0.97) and showed significant positive correlations with self-efficacy at Time 2 (*M* = 3.39, *SD* = 0.35; *r* = 0.298, *p* < 0.01) and positive affect (*M* = 3.49, *SD* = 0.62; *r* = 0.257, *p* < 0.01). In addition, self-efficacy was strongly correlated with positive affect at T3 (*r* = 0.495, *p* < 0.01). In contrast, child-centrism, with a mean of 4.86 (*SD* = 0.98), did not show significant associations with life satisfaction, self-efficacy, or positive affect.

### 3.3. Serial Mediation Models

**Figure 1:**
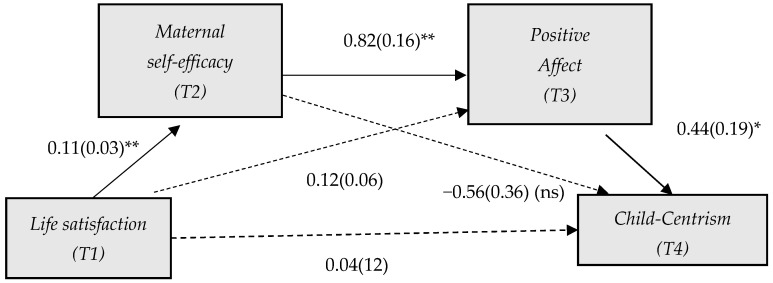
Path diagram depicting the direct and indirect effects linking life satisfaction and child-centrism. Note: A serial multiple mediation analysis was conducted with child-centrism as the outcome variable, life satisfaction as the predictor, and maternal self-efficacy and positive affect serving as the first and second mediators, respectively. The diagram displays unstandardized regression coefficients (with standard errors in parentheses) and their associated *p*-values (ns non-significant, * *p* < 0.05, ** *p* < 0.01). Values in parentheses represent the direct effects, controlling for the mediating pathways. Solid lines indicate statistically significant paths, while dashed lines denote non-significant ones.

A serial mediation analysis was conducted to investigate whether life satisfaction during the third trimester of pregnancy (predictor) influenced the adoption of a child-centrism parenting style five years postpartum (criterion), using two sequential mediators: maternal self-efficacy, assessed eight weeks postpartum, and positive affect, measured five months postpartum. The results showed a significant indirect effect of life satisfaction on child-centrism through the serial pathway (*B* = 0.038, *SE* = 0.025, 95% *CI* = [0.003, 0.101]), suggesting that greater prenatal subjective well-being facilitates greater perceptions of maternal competence, which in turn increases positive affect in the early postpartum period and ultimately translates into greater adoption of a child-centered parenting style (see Figure 1).

However, the simple indirect effects of life satisfaction on child-centrism through self-efficacy (*B* = −0.06, *SE* = 0.45, 95% *CI* = [−0.16, 0.011]) and through positive affect (*B* = 0.49, *SE* = 0.51, 95% *CI* = [−0.017, 0.177]) were not significant. Furthermore, no significant direct effect of the predictor on child-centrism was found (*B* = 0.04; *SE* = 0.12; *t* = 0.30; 95% *CI* = [−0.279, 0.205]; *p* = 0.76). These findings are presented in Figure 1.

## 4. Discussion

The proposed model examines the longitudinal relationship between life satisfaction during the third trimester of pregnancy (T1), maternal self-efficacy eight weeks postpartum (T2), positive affect five months postpartum (T3), and the adoption of a child-centered parenting style five years postpartum (T4). In the Israeli context, where motherhood is considered not only a personal experience but also a pillar of cultural and social identity [51,52,53], these findings are particularly relevant. Pronatalist policies, coupled with an emphasis on family and community ties, provide mothers with a strong support network that, in principle, should enhance their well-being [54]. However, consistent with previous research [55,56], some mothers with insecure attachment styles may be less likely to utilize available community resources, which can contribute to lower levels of life satisfaction. The results show a significant effect of prenatal life satisfaction on maternal self-efficacy, suggesting that greater subjective well-being at the end of pregnancy is associated with a stronger perception of one’s competencies to cope with motherhood, which aligns with previous evidence suggesting that higher life satisfaction enhances psychological preparedness for parenthood and contributes to greater parental maturity [18]. This self-efficacy, in turn, favors an increase in positive affect in the early postpartum period, becoming the most determining factor for the adoption of a child-centered parenting style. Thus, life satisfaction can boost cognitive and affective resources (self-efficacy [57] and positive emotions [58]), which are ultimately reflected in the way mothers prioritize their children’s needs.

Furthermore, a simpler mediation model was evaluated, in which prenatal life satisfaction exerted a direct effect on positive affect, confirming that a mother’s subjective well-being can immediately influence her emotional states during the postpartum period. However, prenatal life satisfaction did not show a direct effect on child-centrism, reinforcing the idea of a sequential process in which, first, self-efficacy is strengthened, then positive affect is enhanced, and, finally, this affective state translates into a more child-centered parenting style. This result is particularly novel and interesting as it raises the need for a series of cognitive–emotional resources that link women’s life satisfaction during pregnancy with child-centrism. Psychological well-being during pregnancy, such as high levels of self-reported life satisfaction and a sense of “flourishing”, is associated with a decreased risk of perinatal depression and anxiety [59]. However, the importance of intermediate variables in this relationship, such as expectations, has also been pointed out [60]. Unmet expectations are important as modifiable risk factors of postpartum anxiety and depression in women [61]. In this line, our study proposes child-centrism as an adaptive parenting style for the mother and the baby, based on the woman’s life satisfaction in the prenatal stage. In line with the previous literature regarding other health outcomes (i.e., anxiety, depression), our results indicate that life satisfaction in the prenatal period is an important predictor of child-centrism, but that maternal expectations (i.e., self-efficacy) play an important role in the development of positive affect in maternity and in the choice of child-centrism as a parenting style within that vital sense.

Importantly, these processes should also be interpreted within the unique sociocultural context characterizing Israeli motherhood. In the Israeli sociocultural context, characterized by dense networks of extended family ties and significant community affiliations, these factors play a central role in shaping maternal experiences. Familism values, which are prominent in Israeli society, emphasize collective responsibility for childrearing and provide emotional and practical support during pregnancy and the early stages of motherhood [62]. Such potential sources of informal social support may contribute to strengthening a mother’s parental self-efficacy [63] by enhancing her confidence in fulfilling the maternal role, as well as by offering positive models of motherhood. Furthermore, strong community ties and social norms surrounding motherhood may foster positive affect by providing social validation and reducing feelings of isolation during the postpartum period. Thus, these sociocultural support systems may reinforce the mediation pathway identified in the present study, whereby prenatal life satisfaction promotes maternal self-efficacy and positive emotional experiences, ultimately contributing to the adoption of a child-centered parenting style.

This longitudinal study presents certain limitations inherent to such design. The dropout rate, although comparable to that of similar research [40,41,42], could introduce bias if the mothers who complete all phases do not represent the general population. Furthermore, it is possible that, over time, changes in the personal and contextual circumstances of the participants have occurred, affecting the stability of the relationships between the variables. Reliance on self-report measures may introduce response bias, particularly for constructs related to maternal self-efficacy and child-centrism, where social desirability concerns might influence reporting. Additionally, while the longitudinal design represents a strength, the predetermined measurement intervals may not optimally capture critical transition periods in maternal adjustment and child development. Although various sociodemographic factors are controlled, other relevant aspects—such as coping mechanisms and variations in social support—are not exhaustively examined. In addition, while the internal consistency of the Child-Centrism Scale was acceptable in this study, its factorial structure was not formally evaluated. Future research could benefit from conducting confirmatory factor analyses (CFAs) on the Child-Centrism Scale in Hebrew-speaking Israeli populations to further strengthen evidence of its structural validity. These restrictions must be weighed considering the complexity involved in conducting long-term follow-up in research of this type [38,39], highlighting the need for future studies with larger samples and more comprehensive follow-up methods to better understand the evolution of maternal well-being.

Despite these limitations, this study has important practical implications. First, the findings suggest that prenatal interventions should focus on strengthening expectant mothers’ life satisfaction, as this translates into greater self-efficacy and an increase in positive affect during the postpartum period, crucial elements for the development of a child-centered parenting style. Prenatal education programs could include workshops focused on emotional management, strategies to strengthen confidence in the maternal role, and group activities that enhance social support. Furthermore, it would be beneficial to develop interventions that identify mothers with insecure attachment styles early on, offering counseling and psychological follow-up that allows them to fully utilize the community and institutional resources available in Israel. Furthermore, in the postpartum period, the implementation of support groups and peer mentoring programs can facilitate the transition to motherhood, fostering an environment that encourages the exchange of experiences and the building of solidarity networks. Furthermore, policies that encourage the continuation of these support over time—such as counseling services and follow-up programs during the early years—could help mitigate the decline in life satisfaction in the long term. Ultimately, these results reinforce the need for comprehensive strategies that combine psychological, educational, and social interventions to improve maternal well-being and, consequently, promote optimal child development.

## 5. Conclusions

In summary, this longitudinal study demonstrates that higher prenatal life satisfaction significantly predicts greater maternal self-efficacy, which in turn enhances positive affect during the early postpartum period. These cognitive and affective resources ultimately contribute to a more child-centered parenting style over time. Although prenatal life satisfaction did not directly influence child-centrism, its indirect effect—transmitted through self-efficacy and positive affect—highlights the importance of early maternal well-being in shaping long-term parenting practices. Within the Israeli sociocultural context, characterized by strong family values and robust pronatalist policies [51,64], these findings underscore the potential of targeted prenatal interventions to foster emotional resilience and effective parenting. Despite limitations such as participant attrition and the potential influence of unmeasured variables (e.g., coping strategies and fluctuating social support), our results emphasize the need for comprehensive, multidisciplinary strategies that integrate psychological, educational, and social interventions. Future research with larger, more diverse samples and more detailed follow-up measures is essential to further clarify these relationships and support the development of interventions aimed at enhancing maternal well-being and, consequently, optimizing child development outcomes.

## Figures and Tables

**Table 1:** Descriptive statistics and correlations among key study variables.

	*M* (*SD*)	*Min*–*Max*	2	3	4
1. Life satisfaction (T1)	5.43 (0.97)	1–7	0.298 **	0.257 **	−0.121
2. Self-efficacy (T2)	3.39 (0.35)	1.83–4		0.495 **	−0.123
3. Positive affect (T3)	3.49 (0.62)	1.40–5			0.100
4. Child-centrism (T4)	4.86 (0.98)	2.14–7			

** *p* < 0.01.

## Data Availability

The data presented in this study are available on request from the corresponding author. The data are not publicly available due to privacy restrictions.
